# Explore the Interaction between Ellagic Acid and Zein Using Multi-Spectroscopy Analysis and Molecular Docking

**DOI:** 10.3390/foods11182764

**Published:** 2022-09-08

**Authors:** Shunan Zhao, Yong Deng, Tianyi Yan, Xiaoling Yang, Weidong Xu, Donghong Liu, Wenjun Wang

**Affiliations:** 1 National-Local Joint Engineering Laboratory of Intelligent Food Technology and Equipment, Zhejiang Key Laboratory for Agro-Food Processing, Zhejiang Engineering Laboratory of Food Technology and Equipment, Fuli Institute of Food Science, College of Biosystems Engineering and Food Science, Zhejiang University, Hangzhou 310058, China; 2 School of Liquor and Food Engineering, Guizhou University, Guiyang 550000, China; 3 Ningbo Research Institute, Zhejiang University, Ningbo 315100, China; 4 Innovation Center of Yangtze River Delta, Zhejiang University, Jiashan 314100, China

**Keywords:** ellagic acid, zein, interaction, spectroscopic analysis, non-covalent binding, molecule docking simulation

## Abstract

With the increasing interest in value-added maize products, the interaction of zein with bioactive molecules to become more nutritional and beneficial to human health has gained a lot of attention. To broaden the application of ellagic acid (EA) in maize flour products, we investigated the interaction between zein and EA. The fluorescence quenching type of zein interacting with EA was mainly static quenching through hydrophobic interaction, as demonstrated by quenching behavior modeling, and ultraviolet-visible spectroscopy confirmed the formation of zein–EA complexes. Synchronous fluorescence spectroscopy showed that EA reduced the polarity of zein around tyrosine residues, which were exposed to a more hydrophobic microenvironment. Meanwhile, circular dichroism suggested that EA noticeably changed the secondary structure of zein, which was mainly reflected in the increase of α-helix and β-sheet content and the decrease of random coil content. Finally, the molecular docking simulation found that zein could have five active sites binding to EA and there was hydrogen bond interaction besides hydrophobic interaction. The findings of this study provided a basis for a theory for the interaction mechanism between zein and EA, which could be essential for developing value-added plant-derived protein products using EA as a functional component.

## 1. Introduction

There is a growing interest in the development of innovations in plant-based foods due to their low environmental impact and many health benefits. Distinguished from animal proteins, plant proteins that are free of cholesterol and emit less carbon dioxide are generally recognized as safe (GRAS) and good for people who are vegetarian. Meanwhile, plant proteins were more abundant and cheaper than animal proteins and had unique functional properties and nutritional characteristics such as reducing the risk of type 2 diabetes and cardiovascular disease. In recent years, plant proteins have been increasingly replacing animal proteins. In particular, zein was a vital protein. Zein is the by-product of maize starch production, accounting for 40% of total protein content, which is a relatively cheap and nutritious vegetable protein.

Proteins and polyphenols are two essential components in complex food systems, and their interactions are inevitable during food production, food processing, and storage. Previous research has demonstrated that polyphenols possess good binding affinities to proteins, which alter their structures and functional properties [1]. Furthermore, the interaction between food polyphenols and protein would affect food texture, safety, function, as well as nutrition. 

The interactions between proteins and polyphenols include non-covalent and covalent interactions. Unlike the non-covalent interaction, the covalent interaction of polyphenol and protein must be generated under specific conditions such as an alkaline environment or a redox pair of initiator systems induced by ascorbic acid and hydrogen peroxide [2] or enzyme oxidation [3]. During the covalent interaction process, quinones and semiquinone radicals were produced through Michael addition, which has been proven to be related to a series of pathological disorders in vivo [4]. However, the non-covalent interactions between polyphenols and proteins were more likely to occur under moderate conditions. It could be attributed to hydrophobic interaction [5,6], hydrogen bonding [7,8], electrostatic interaction, and van der Waals forces, and the resulting complexes would have fewer safety issues than the covalent ones [9]. 

Zein accounted for almost half of the total endosperm protein of maize and belonged to the class of prolamins [10]. As a valuable byproduct of the corn milling industry, it could be edible, biodegradable, environmentally friendly, non-toxic, and biocompatible [11,12]. Containing hydrophilic and hydrophobic regions on the surface, zein could be prone to converting into spherical colloidal nanoparticles, which usually contributes to the targeted and controlled delivery of natural active ingredients in food, biological, and pharmaceutical fields [13,14]. Meanwhile, proteins that interacted with food polyphenols would obtain better antioxidant activity. For example, both zein-(-)-epigallocatechin gallate (EGCG) complexes and zein–quercetagetin complexes have been reported to perform better antioxidant activity than raw protein [15]. Moreover, zein could protect bioactive compounds from a harsh gastric environment due to its resistance to digestive enzymes [16]. Zein, therefore, has been attracting considerable interest in the encapsulation and delivery of bioactive compounds.

Ellagic acid (EA), a kind of tannin that belongs to the natural polyphenol compound, could be recovered from various fruits and nuts processing waste for valorization, including pomegranate peel [17], raspberry wine pomace [18], chestnut shells [19], and longan seeds [20]. It has pronounced pharmacological properties such as antioxidant [21], anticancer [22], anti-inflammatory [23], and anti-atherosclerosis activity [24], and treatment for diabetes [25] and neurodegenerative diseases [26]. Structurally, EA, a trans gallic acid dimer derivative, comprises a dilactone of hexahydroxydiphenic acid (HHDP), characterized by four fused rings and four hydroxyl groups. Because of the above structure, EA has an amphipathic property, possessing strong protein affinity [27]. The distinctive structure enhances the ability of EA to accept electrons, hence leading to a strong antioxidant capacity [28]. Moreover, it has been proven that the interaction between EA and protein could cause structural changes in the protein. It was found that the interaction of EA and gallic acid at a high concentration (500 μM) with bovine serum albumin (BSA) resulted in an unfolding of the secondary structure of BSA as revealed by circular dichroism spectra (CD). The binding constants indicated a pH-dependent binding of phenolic acids by BSA [29]. The previous study found that EA effectively quenched the intrinsic fluorescence of human serum albumin (HSA) by static quenching, and hydrophobic and hydrogen bond interactions played essential roles in stabilizing the HSA–EA complex. CD spectroscopic data indicated that EA altered the secondary structure of HSA with a decreased fraction of alpha helicity [30]. Regarding gluten, EA could decrease α-helix content of gluten protein, which was analyzed through Raman spectroscopy [31]. In addition to affecting the structure, EA could also influence the properties of proteins when binding with the protein. By comparison with the amyloid-like aggregates of ovalbumin caused by heat, the surface hydrophobicity of the ovalbumin–EA complex significantly declined. With an improved soluble multimer structure, the non-covalent interaction enabled ovalbumin to be applied to the beverage industry [32]. A recent study found that after interacting with EA, the α-lactalbumin–EA complex had superior antioxidant capacity than EA alone [33].

However, the interaction of EA with zein has not been studied so far. The objectives of the study are (1) to investigate the fluorescence quenching mechanism between EA and zein; (2) to elucidate the binding mechanism of EA with zein by molecular docking simulation; (3) to characterize the conformational changes of zein after interacting with EA. Our work could provide the theoretical basis for applying EA in protein products and novel insights into developing protein–polyphenol binary complexes with potentially improved functions.

## 2. Materials and Methods

### 2.1. Materials

EA (purity >99%) was purchased from Shanghai Macklin Biochemical Co., Ltd. (Shanghai, China). Zein (purity >98%) was purchased from J&K Scientific Co., Ltd. (Beijing, China). Ethanol was purchased from Merck Chemical Technology (Shanghai) Co., Ltd. (Shanghai, China), which was of HPLC grade. 

EA was dissolved in 50% methanol aqueous solution to obtain 1 mg/mL stock solution, and then a dilution was performed to get the required gradient concentration, and the dilute solutions were kept at 4 °C. 0.05 mg/mL zein solution was prepared in 70% ethanol aqueous solution and kept at 4 °C.

### 2.2. Ultraviolet-Visible (UV-Vis) Spectroscopy Analysis

Samples of 50 μL of different EA gradient concentrations (0, 0.1, 0.2, 0.3, 0.4, 0.5, 0.6, 0.7, 0.8, 0.9, 1.0 mg/mL) were added dropwise into 4 mL zein solution (0.05 mg/mL) in triplicate at 25 °C. The UV–Vis spectra of the specimens were acquired according to a previous method with a slight modification [34]. Briefly, the UV–Vis absorbance of zein solution containing different EA concentrations was recorded from 200 to 400 nm by a ultraviolet-visible spectrophotometer (UV-2550, Shimadzu Inc., Kyoto, Japan).

### 2.3. Fluorescence Spectra Analysis

The determination of fluorescence spectra was carried out by using a Cary eclipse fluorescence spectrometer (Varian Inc., Los Angeles, CA, USA). The different mixtures used above were heated in a water bath with a constant temperature of 18 °C (291 K), 25 °C (298 K), and 37 °C (310 K) for 5 mins, respectively. The intrinsic fluorescence spectra were obtained in the 300–500 nm wavelength range under 280 nm excitation. Both the excitation and emission slit widths were 5 nm. Meanwhile, synchronous fluorescence spectra were measured at a wavelength interval (Δ λ) of 15 nm at 25 °C.

### 2.4. Quenching Behavior Modeling

Quenching has a variety of mechanisms, usually including dynamic quenching and static quenching. When a collision between the fluorescent substances and quencher occurs, dynamic quenching would occur. Different from the dynamic quenching, the fluorophore forms a ground-state non-fluorescent complex with the quencher, resulting in static quenching. The two types of quenching could be well discriminated by their varying dependence on viscosity and temperature. The Stern–Volmer equation (Equation (1)) was usually utilized to judge the type of quenching by the modeling function in Origin 2019 [35].

(1) $$\frac{F_{0}}{F}=1+{\;K}_{q}\tau_{0}\left[Q\right]=1+{\;K}_{\mathrm{sv}}\left[Q\right]$$

where F_0_ and F are the fluorescence intensity of the original fluorescent substance with a quencher (phenolic acids et al.) free and with a quencher, respectively; K_q_ represents the quenching rate constant of the phenolic acid; τ_0_ represents the lifespan of the fluorophore without quencher and the average value is about 10^−8^ s; K_sv_ represents the dynamic quenching constant; Q represents the concentration of quencher.

The Stern–Volmer equation with modification (Equation (2)) was utilized to determine the binding site number and binding affinity by the modeling function in Origin 2019.

(2) $$\mathrm{lg}\frac{F_{0}\;-\;F}{F}={\;\mathrm{lgK}}_{A}+\;\mathrm{nlg}\left[Q\right]$$

where F_0_ is the fluorescence intensity of the original fluorescent substance without a quencher, F represents the fluorescence intensity with the quencher; Q represents the concentration of quencher; K_A_ represents the binding affinity; n represents the number of the binding site.

### 2.5. Thermodynamic Parameters

Thermodynamic parameters and force types of interaction could be determined by van’t Hoff equation (Equations (3) and (4)) [36]:

(3) $$\ln\left(K_{2}{/K}_{1}\right)=\left[\frac{1}{T_{1}}-\frac{1}{T_{2}}\right]\Delta H/R$$

(4) ΔG=ΔH − TΔS=-RTlnK

where R represents the gas state constant, which is 8.314 J/(K·mol); T represents the temperature; K represents the binding constant; ΔH represents enthalpy changes; ΔS represents entropy changes; ΔG represents Gibbs free energy changes.

### 2.6. Circular Dichroism (CD) Spectroscopy Determination

A J-1500 spectropolarimeter with a PM-539 detector (JASCO Inc., Tokyo, Japan) was utilized to determine CD spectra. The CD determination of zein protein solution without and with EA (different concentrations) in a cell of 1 mm light length was recorded from 190 nm to 260 nm at a scanning rate of 100 nm/min at 20 °C. The secondary structure of the protein was analyzed by using the dichroweb database.

### 2.7. Molecular Docking Simulation

The simulation approach was employed to research the interaction between zein and ellagic acid at a single-molecule level. The protein crystal structure utilized for autodock was acquired from the PDB database. Zein protein was obtained by a Swiss model server (Swissmodel.expasy.org) based on sequence homology modeling with UniProt ID P04706. The 3D structure of ellagic acid was obtained from the PUBCHEM database, and then energy minimization was carried out by Chem 3D V20 under the MMFF94 force field.

In our work, Autodock vina software (version 1.1.2, originally designed and implemented by Dr. Oleg Trott in the Molecular Graphics Lab at The Scripps Research Institute (La Jolla, CA, USA)) was utilized for molecular docking simulation. First of all, to remove water molecules, salt ions, and other small molecules, PyMol software (version 2.5, a user-sponsored molecular visualization system on an open-source foundation, maintained and distributed by Schrödinger Platform (New York, NY, USA)) was applied to deal with all of the target proteins. Secondly, a docking box was constructed and PyMol was used to determine the center of the docking box based on the position of the crystal ligand. The side length of the box was set to 75 angstroms to wrap the whole protein completely. Moreover, ADFRsuite (version 1.0) was used to convert all of the pretreated ligands and target proteins into PDBQT format, which matched with molecular docking software. The detailed degree of the global search was set to 32, and other parameters remained the default settings under docking. The desired docking conformation with the highest simulation score was chosen to be considered as the best binding one. Lastly, we utilized PyMol 2.5 for visual analysis of the best docking results.

### 2.8. Statistical Analysis

All tests were implemented in triplicate and values were reported as the mean ± standard deviation. Data were processed by one-way variance (ANOVA) to analyze the results, and the significance among samples were calculated by using Duncan’s test with SPSS 20.0 (SPSS Inc., Chicago, IL, USA). Significance was considered as *p* < 0.05.

## 3. Results and Discussion

### 3.1. Effect of EA on UV–Vis Spectra of Zein

**Figure 1:**
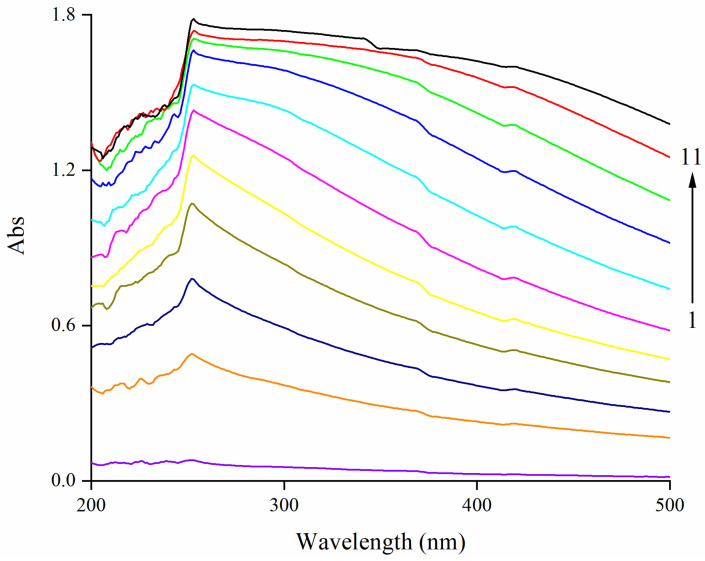
Effect of EA on the UV–Vis spectroscopy of zein. Note: EA: ellagic acid; the concentration of zein is 0.05 mg/mL; curve 1: violet curve (0 mg/mL EA); 2: orange curve (0.1 mg/mL EA); 3: navy curve (0.2 mg/mL EA); 4: dark yellow curve (0.3 mg/mL EA); 5: yellow curve (0.4 mg/mL EA); 6: magenta curve (0.5 mg/mL EA); 7: cyan curve (0.6 mg/mL EA); 8: blue curve (0.7 mg/mL EA); 9: green curve (0.8 mg/mL EA); 10: red curve (0.9 mg/mL EA); 11: black curve (1.0 mg/mL EA).

UV–Vis spectroscopy is widely used to study the interaction between small molecules and biopolymers. Briefly, the change of the peak position was usually considered as the conformational change caused by the change in the microenvironment of hydrophobic amino acid residues in protein, and the change of the absorbance intensity was a sign of the strength change of the interaction [37]. The UV–Vis spectra showed that the intensity peak of zein moved from originally 250 nm with a red shift to 253 nm with higher EA concentrations (Figure 1), suggesting the conformational change of zein. The red shift phenomenon could be attributed to the increase in the exposure of Tyr residues with higher contents of hydrophobic and chromophore groups in the modified conformation of zein after adding EA. A similar red shift in UV–Vis spectra was also found in the interaction between theasinesin and BSA [38], indicating the existing interaction between polyphenol and protein. Regarding the absorbance intensity, a significant upward trend could be seen with the increase in EA concentration, which was dose-dependent, confirming the enhanced interaction between zein and higher concentrations of EA.

### 3.2. Effect of EA on Intrinsic Fluorescence Spectrum of Zein

#### 3.2.1. The Quenching Effects of EA on the Fluorescence Emission Intensity of Zein

**Figure 2:**
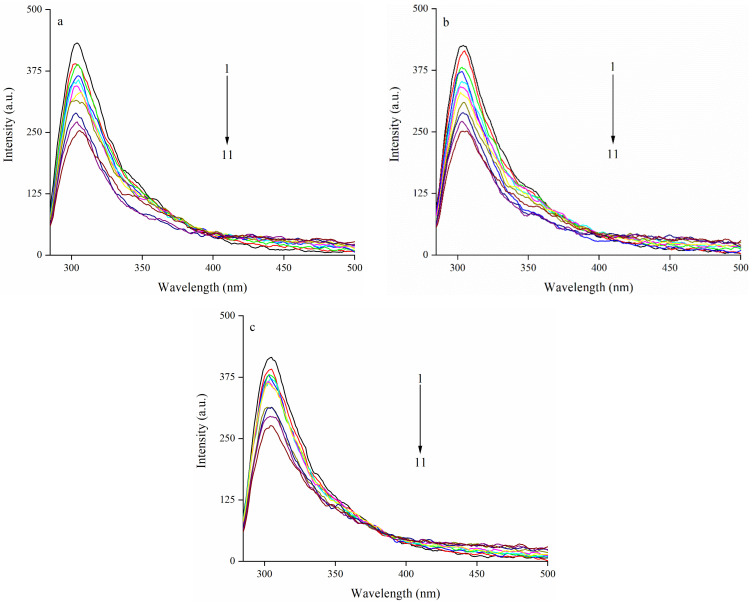
Effect of EA on the fluorescence emission intensity of zein. Note: (**a**–**c**) represents different treatment temperature for EA and zein; (**a**): 18 °C (291 K); (**b**): 25 °C (298 K); (**c**): 37 °C (310 K); the concentration of zein is 0.05 mg/mL. EA: ellagic acid; curve 1: black curve (0 mg/mL EA); 2: red curve (0.1 mg/mL EA); 3: green curve (0.2 mg/mL EA); 4: blue curve (0.3 mg/mL EA); 5: cyan curve (0.4 mg/mL EA); 6: magenta curve (0.5 mg/mL EA); 7: yellow curve (0.6 mg/mL EA); 8: dark yellow curve (0.7 mg/mL EA); 9: navy curve (0.8 mg/mL EA); 10: purple curve (0.9 mg/mL EA); 11: wine curve (1.0 mg/mL EA).

The intrinsic fluorescence is a valuable technique to gain insight into the interaction between protein and polyphenol [39,40], which is mainly influenced by Trp and Tyr, which are the two most important fluorophores of protein. Figure 2a–c show the influence of EA on the fluorescence emission intensity of zein at different treatment temperatures. According to Figure 2, for all three temperatures, the reduction in the fluorescence emission intensity was induced with the increase of EA concentration (from 0 to 1 mg/mL), indicating a more significant fluorescence quenching effect of zein, which was dose-dependent. Similarly, after combining with resveratrol, the fluorescence emission intensity of zein steadily declined as the resveratrol concentration increased [41]. Additionally, a similar result was also found in that a higher (-)-epigallocatechin-3-gallate (EGCG) concentration caused lower fluorescence emission intensity when mixing with SPI [42]. 

Since being sensitive to the polarity change of the microenvironment during transformation, the intrinsic fluorescence of Trp, Tyr, and Phe residues in proteins has already been proven to be a good way to determine the change of the tertiary structure. The maximum emission wavelength of zein was blue-shifted after EA was added, corresponding to a more hydrophobic microenvironment of Tyr [43]. Moreover, it was also observed that the same shift in the maximum emission wavelength of zein came about after an interaction with ferulic acid, suggesting a similar change in the microenvironment around Tyr [44]. 

#### 3.2.2. Fluorescence Quenching Behavior Modeling

**Figure 3:**
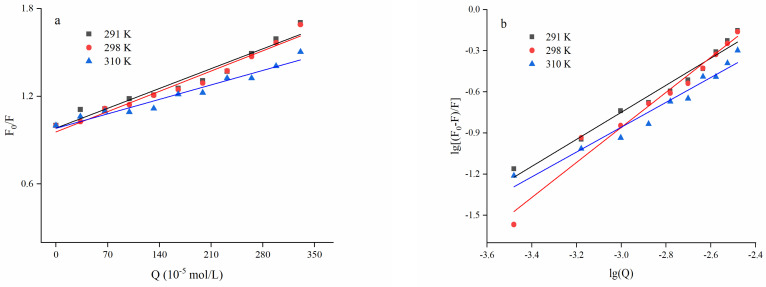
The plot for quenching on zein by EA at different temperatures. Note: (**a**): the Stern–Volmer plots; (**b**): the lg[(F_0_ − F)/F] vs. lg(Q) plots for the binding of EA with zein; EA: ellagic acid; the concentration of zein is 0.05 mg/mL; EA concentration is 0, 0.1, 0.2, 0.3, 0.4, 0.5, 0.6, 0.7, 0.8, 0.9 and 1.0 mg/mL, respectively.

Table 1 shows the quenching rate constants and determination coefficients (R^2^) calculated by the Stern–Volmer formula exhibited in Section 2.4. The determination coefficients from all three temperatures were close to 1, indicating an excellent linear fit reduction of the fluorescence emission intensity with increased EA concentration, as shown in Figure 3a. This conformed to the existence of varying fluorophores in zein with affinity to EA. It was found that after adding EA to BSA, a linear reduction of the fluorescence emission intensity also occurred, showing that EA could strongly interact with BSA [29]. For biopolymers, the maximal quenching rate constant of varied quenchants produced by diffusion collision was 2 × 10^10^ L/(mol·s) [45,46]. The fluorescence quenching rate (K_q_) of EA on zein (1.4 × 10^11^ L/(mol·s)–2.0 × 10^11^ L/(mol·s)) was higher than the maximal value controlled by diffusion collision, which further indicated the way of quenching was the static type caused by the intermolecular forces to form a complex instead of the dynamic type caused by diffusion collision. However, there was a dynamic quenching between zein and resveratrol [41], indicating that the type of polyphenol could affect the quenching type.

For the static quenching type, a modified Stern–Volmer equation (Equation (2)) could be utilized to calculate the binding constant (K_A_) and the number of binding sites (n). As shown in Table 2 and Figure 3b, the K_A_ (from 1.63 × 10^2^ to 1.27 × 10^3^) increased with the increasing temperature, which indicated that the binding affinity between EA and zein became stronger. The binding constants found for zein and EA was similar to those reported for other proteins [47,48]. The results also found that the binding site was close to 1 (from 0.9065 to 1.2786) at all three temperatures, indicating that EA and zein were bound according to a molar ratio of 1:1. Similarly, EGCG was stirred with zein solution, and the binding site number was about 1. This meant that only a single binding site was involved in the binding process [49].

#### 3.2.3. Thermodynamic Parameters and Type of Quenching Force

The thermodynamic parameters calculated by Equations (3) and (4) are shown in Table 3. The quenching force types between EA and zein would be calculated by the van’t Hoff formula. The main quenching forces between low molecular weight molecules and biopolymers include hydrogen bond, hydrophobic action, electrostatic action, and van der Waals force. Consisting of enthalpy change (ΔH) and entropy change (ΔS), thermodynamic parameters are always utilized to judge the type of the major non-covalent forces as the following: (a) if both △H and △S are positive, it is the hydrophobic effect; (b) if both ΔH and ΔS are negative, it is mainly van der Waals force and hydrogen bond action; (c) if ΔH is negative while ΔS is positive, it is electrostatic action [34]. It can be seen from Table 3 that all of the conditions showed positive ΔH and ΔS, so the type of quenching force involved in zein–EA is hydrophobic interaction. Previous studies suggested hydrophobic interactions mainly occurred between hydrophobic areas of proteins and aromatic rings of polyphenols [50]. Additionally, negative ΔG values suggested that the combination process of EA and zein could be spontaneous. Positive ΔH values showed that the interaction was endothermic and heating was favorable for the reaction, concurring with the increase of K_A_ induced by the increased temperature (Table 2).

### 3.3. Effect of EA on Synchronous Fluorescence Spectra of Zein

**Figure 4:**
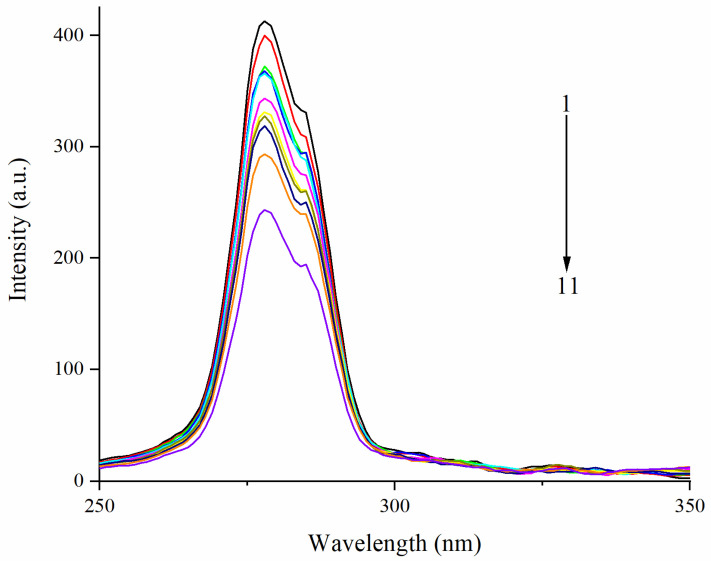
Effect of EA on synchronous spectra of zein. Note: effect of EA on synchronous spectra of zein with Δλ = 15 nm; EA: ellagic acid; the concentration of zein is 0.05 mg/mL; curve 1: black curve (0 mg/mL EA); 2: red curve (0.1 mg/mL EA); 3: green curve (0.2 mg/mL EA); 4: blue curve (0.3 mg/mL EA); 5: cyan curve (0.4 mg/mL EA); 6: magenta curve (0.5 mg/mL EA); 7: yellow curve (0.6 mg/mL EA); 8: dark yellow curve (0.7 mg/mL EA); 9: navy curve (0.8 mg/mL EA); 10: orange curve (0.9 mg/mL EA); 11: violet curve (1.0 mg/mL EA).

Synchronous fluorescence spectra could provide information about the microenvironment in the proximity of chromophore molecules. Because of the shortage of Trp residues in zein [51], the synchronous spectra were only performed in the condition of the wavelength interval set at 15 nm to research the influence of EA on the microenvironment of the Tyr residue of zein [52]. Synchronous fluorescence spectra of zein and the complex upon adding EA are shown in Figure 4. With the addition of EA, the fluorescence intensity gradually decreased. The maximum emission wavelength of Tyr was blue-shifted in zein, indicating that the hydrophobicity of the microenvironment in the vicinity of the Tyr residues increased. Similarly, there was blue-shifted at the Δλ  =  15 nm in the myofibrillar protein-chlorogenic acid interacting system, which also meant the hydrophobic environment surrounding Tyr residues [53]. Contrary to our result, the polarity around Tyr residues increased and exposed them to a more hydrophilic microenvironment with a red shift of the maximum emission wavelength [54]. Therefore, all the above results verified the conformation change of zein induced by the EA. 

### 3.4. Effect of EA on Conformational Changes of Zein Determined by CD Spectroscopy

**Figure 5:**
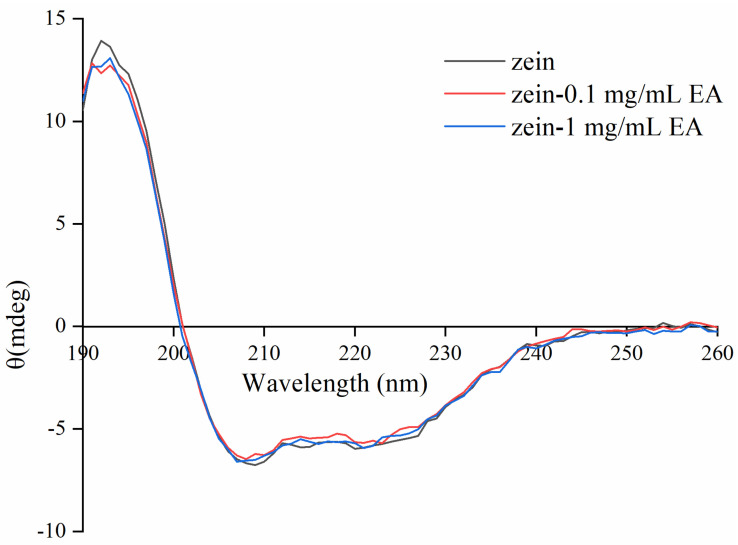
Effect of EA on the circular dichroism spectroscopy of zein. Note: EA: ellagic acid; the concentration of zein is 0.05 mg/mL; zein−0.1 mg/mL EA: 0.05 mg/mL zein reacted with 0.1 mg/mL EA; zein−1mg/mL EA: 0.05 mg/mL zein reacted with 1 mg/mL EA.

To clarify the influence of EA on the secondary structure of zein, we employed CD spectroscopy for analysis (Figure 5). The conformational changes of all samples are shown in Table 4. It was found that zein contained 51.12% α-helix, 12.51% β-sheet, 16.91% β-turn, and 19.46% random coil. With the addition of EA, the contents of α-helix and β-sheet increased, while β-turn and random coil decreased. Previous studies found α-zein to be present in the form of an extended oblong structure in ethanol aqueous solution [55]. Consequently, the distinctive configuration of the protein in the medium (aqueous ethanol solution) would boost the accessibility to the reactive sites of zein for EA. The CD spectroscopy of zein illustrated the typical features of α-helix, which possessed two strong negative ellipticity values at 208 nm and 222 nm, and there were two negative Cotton effects (deflection occurred when linearly polarized light passed through optically active substances) in the range of 200–250 nm, which were W-shaped. The above results demonstrated that the polarity of amino acid residues microenvironment was transformed, resulting in the conformation change of zein.

### 3.5. Molecular Docking Simulation

Molecular docking simulation technology is an effective and convenient tool to give a deep insight into the interactions between small molecules and targets and their binding energies. The 3D structure model of zein in the study was composed of a helix and a loop, showing a V-shaped structure, which conformed to the previously documented structure models [56]. The docking simulation was analyzed between EA and zein. Generally, when the negative binding affinity energy value was less than −6 kcal/mol, binding was more likely to be induced. In the current study, the binding affinity energy value was −6.8 kcal/mol, which meant that EA had a potential active binding effect on zein. Compared with the binding affinity energy value in other studies, the one for zein and EA was smaller than around −4 kcal/mol for the interaction between zein and EGCG [49], but close to −7.7 kcal/mol for the interaction with curcumin [57]. This may be due to the higher hydrophobicity of EA and curcumin than EGCG.

**Figure 6:**
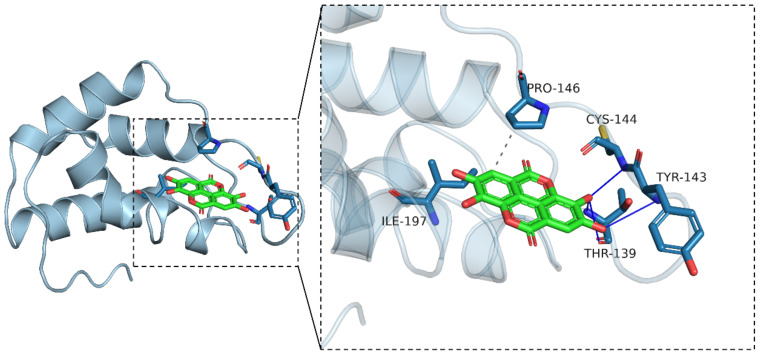
The 3D image of molecular docking for the non-covalent binding of zein with EA. Note: the light blue cartoon is protein; the green stick is the small molecule (EA); the blue line represents hydrogen bond; the gray dashed line represents hydrophobic effect.

The docking location between EA and zein is shown in Figure 6. The light blue cartoon was the zein, and the green stick was EA. Moreover, the blue line represented the hydrogen bond, and the gray dashed line represented the hydrophobic effect. The interaction diagram clearly showed that EA was inserted into the active sites of the zein, where it interacted with many amino acids. The active amino acids within the active sites were Cys 144, Tyr 143, Thr 139, Pro 146, and Ile 197. Cys 144, Tyr 143, and Thr 139 formed hydrogen bonds with EA, while Pro 146 and Ile 197 constituted a hydrophobic pocket to envelop EA. The molecular docking simulation results corresponded to the results of the fluorescence spectrum analysis reported above.

## 4. Conclusions

In our work, the interaction between EA and zein was researched by multi-spectroscopy and molecular docking simulation. The result showed a quenching effect of the intrinsic fluorescence of zein induced by EA, and the effect was strengthened with the increase in EA concentration. It was found that static quenching played a dominant role during the process, which was mainly caused by the hydrophobic interaction. Molecular docking simulation showed five active sites of zein, indicating the existing hydrophobic interaction as well as the hydrogen bond interaction. Moreover, synchronous fluorescence spectra analysis showed the microenvironment of the Tyr residue of zein became more hydrophobic after interacting with EA. The CD spectra revealed that EA had a significant impact on the α-helix and β-sheet contents, indicating great changes in the secondary structure of zein. Although we elucidated the structure change of zein after the interaction, we did not figure out how this altered structure would affect the various properties of zein. Therefore, future studies could focus on the physicochemical properties, antioxidant activity, and stability of zein–EA complexes used in food systems.

## Figures and Tables

**Table 1:** Quenching rate constants (K_sv_) and determination coefficients (R^2^) of EA and zein.

T (K)	K_sv_ (L/mol)	K_q_ (L/(mol·s))	R^2^
291	1.90 × 10^3^	1.90 × 10^11^	0.9529
298	2.00 × 10^3^	2.00 × 10^11^	0.9666
310	1.40 × 10^3^	1.40 × 10^11^	0.9557

EA: ellagic acid; K_sv_ is the dynamic quenching constant; K_q_ is the quenching rate constant of the biomolecule.

**Table 2:** Apparent binding constants (K_A_), binding sites numbers (n), and determination coefficients (R^2^) of EA and zein.

T (K)	n	K_A_ (L/mol)	R^2^
291	0.9871	1.63 × 10^2^	0.9713
298	1.2786	9.47 × 10^2^	0.9740
310	0.9065	1.27 × 10^3^	0.9560

EA: ellagic acid; K_A_ is the binding constant.

**Table 3:** Thermodynamic parameters for interaction between EA and zein.

T (K)	ΔH (kJ/mol)	ΔS (J/(mol•K))	ΔG (kJ/mol)
291	181.28	0.67	−12.32
298	181.28	0.67	−16.98
310	181.28	0.67	−26.42

EA: ellagic acid.

**Table 4:** Fractions of secondary structure of zein in the absence and presence of EA.

Sample	α-helix	β-sheet	β-turn	Random Coil
Zein	51.12 ± 0.83 ^b^	12.51 ± 0.98 ^c^	16.91 ± 0.77 ^a^	19.46 ± 0.58 ^a^
Zein-0.1 mg/mL EA	53.55 ± 0.50 ^a^	16.35 ± 0.39 ^b^	14.82 ± 0.96 ^b^	15.28 ± 0.19 ^b^
Zein-1 mg/mL EA	55.82 ± 0.90 ^a^	19.52 ± 0.36 ^a^	13.36 ± 0.73 ^b^	11.30 ± 0.65 ^c^

EA: ellagic acid; Zein-0.1 mg/mL EA: 0.05 mg/mL Zein reacted with 0.1 mg/mL EA; Zein-1 mg/mL EA: 0.05 mg/mL Zein reacted with 1 mg/mL EA. Different letters are significantly different (*p* < 0.05).

## Data Availability

The data presented in this study are available on request from the corresponding author.
